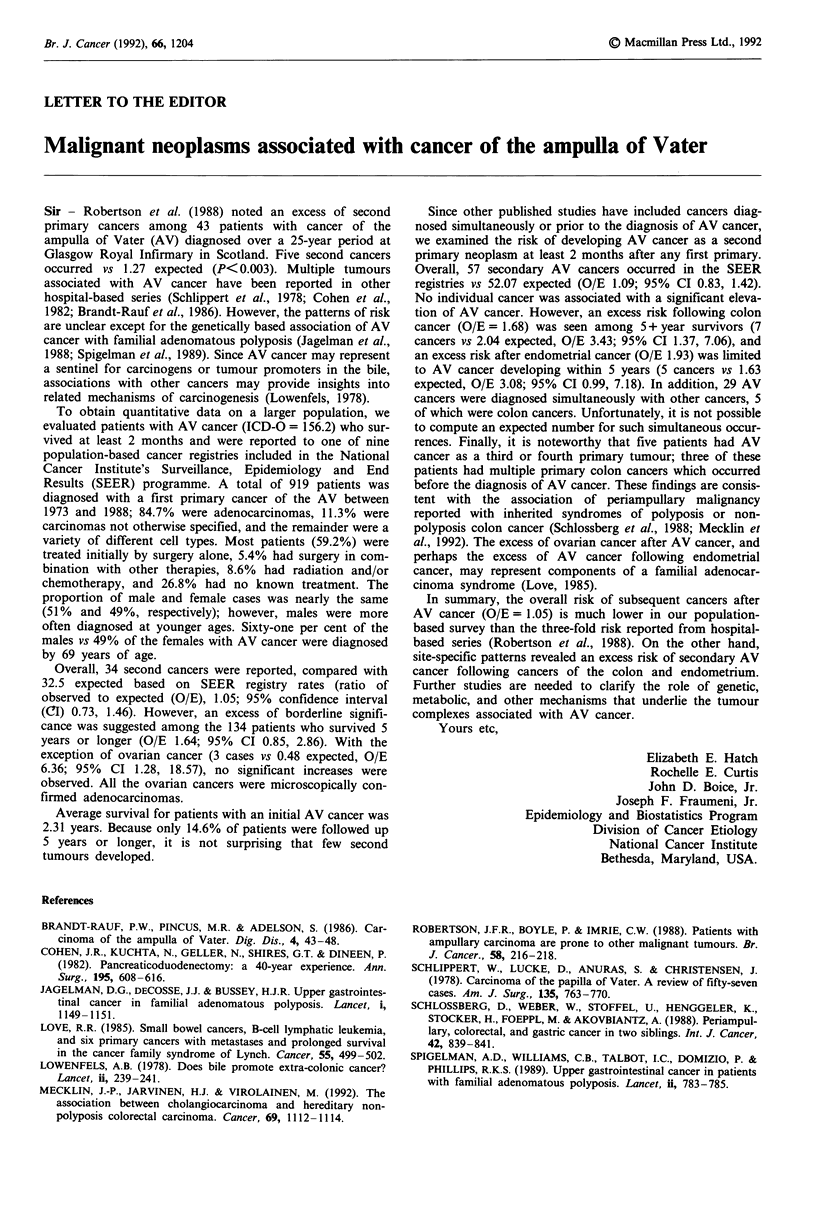# Malignant neoplasms associated with cancer of the ampulla of Vater.

**DOI:** 10.1038/bjc.1992.439

**Published:** 1992-12

**Authors:** E. E. Hatch, R. E. Curtis, J. D. Boice, J. F. Fraumeni


Br. J. Cancer (1992), 66, 1204 © Macmillan Press Ltd., 1992

LETTER TO THE EDITOR

### Malignant neoplasms associated with cancer of the ampulla of Vater

**Sir** – Robertson *et al.* (1988) noted an excess of second primary cancers among 43 patients with cancer of the ampulla of Vater (AV) diagnosed over a 25-year period at Glasgow Royal Infirmary in Scotland. Five second cancers occurred *vs* 1.27 expected ($P<0.003$). Multiple tumours associated with AV cancer have been reported in other hospital-based series (Schlippert *et al.*, 1978; Cohen *et al.*, 1982; Brandt-Rauf *et al.*, 1986). However, the patterns of risk are unclear except for the genetically based association of AV cancer with familial adenomatous polyposis (Jagelman *et al.*, 1988; Spigelman *et al.*, 1989). Since AV cancer may represent a sentinel for carcinogens or tumour promoters in the bile, associations with other cancers may provide insights into related mechanisms of carcinogenesis (Lowenfels, 1978).

To obtain quantitative data on a larger population, we evaluated patients with AV cancer (ICD-O = 156.2) who survived at least 2 months and were reported to one of nine population-based cancer registries included in the National Cancer Institute's Surveillance, Epidemiology and End Results (SEER) programme. A total of 919 patients was diagnosed with a first primary cancer of the AV between 1973 and 1988; 84.7% were adenocarcinomas, 11.3% were carcinomas not otherwise specified, and the remainder were a variety of different cell types. Most patients (59.2%) were treated initially by surgery alone, 5.4% had surgery in combination with other therapies, 8.6% had radiation and/or chemotherapy, and 26.8% had no known treatment. The proportion of male and female cases was nearly the same (51% and 49%, respectively); however, males were more often diagnosed at younger ages. Sixty-one per cent of the males *vs* 49% of the females with AV cancer were diagnosed by 69 years of age.

Overall, 34 second cancers were reported, compared with 32.5 expected based on SEER registry rates (ratio of observed to expected (O/E), 1.05; 95% confidence interval (CI) 0.73, 1.46). However, an excess of borderline significance was suggested among the 134 patients who survived 5 years or longer (O/E 1.64; 95% CI 0.85, 2.86). With the exception of ovarian cancer (3 cases *vs* 0.48 expected, O/E 6.36; 95% CI 1.28, 18.57), no significant increases were observed. All the ovarian cancers were microscopically confirmed adenocarcinomas.

Average survival for patients with an initial AV cancer was 2.31 years. Because only 14.6% of patients were followed up 5 years or longer, it is not surprising that few second tumours developed.

Since other published studies have included cancers diagnosed simultaneously or prior to the diagnosis of AV cancer, we examined the risk of developing AV cancer as a second primary neoplasm at least 2 months after any first primary. Overall, 57 secondary AV cancers occurred in the SEER registries *vs* 52.07 expected (O/E 1.09; 95% CI 0.83, 1.42). No individual cancer was associated with a significant elevation of AV cancer. However, an excess risk following colon cancer (O/E = 1.68) was seen among 5+ year survivors (7 cancers *vs* 2.04 expected, O/E 3.43; 95% CI 1.37, 7.06), and an excess risk after endometrial cancer (O/E 1.93) was limited to AV cancer developing within 5 years (5 cancers *vs* 1.63 expected, O/E 3.08; 95% CI 0.99, 7.18). In addition, 29 AV cancers were diagnosed simultaneously with other cancers, 5 of which were colon cancers. Unfortunately, it is not possible to compute an expected number for such simultaneous occurrences. Finally, it is noteworthy that five patients had AV cancer as a third or fourth primary tumour; three of these patients had multiple primary colon cancers which occurred before the diagnosis of AV cancer. These findings are consistent with the association of periampullary malignancy reported with inherited syndromes of polyposis or non-polyposis colon cancer (Schlossberg *et al.*, 1988; Mecklin *et al.*, 1992). The excess of ovarian cancer after AV cancer, and perhaps the excess of AV cancer following endometrial cancer, may represent components of a familial adenocarcinoma syndrome (Love, 1985).

In summary, the overall risk of subsequent cancers after AV cancer (O/E = 1.05) is much lower in our population-based survey than the three-fold risk reported from hospital-based series (Robertson *et al.*, 1988). On the other hand, site-specific patterns revealed an excess risk of secondary AV cancer following cancers of the colon and endometrium. Further studies are needed to clarify the role of genetic, metabolic, and other mechanisms that underlie the tumour complexes associated with AV cancer.

Yours etc,

Elizabeth E. Hatch
Rochelle E. Curtis
John D. Boice, Jr.
Joseph F. Fraumeni, Jr.
Epidemiology and Biostatistics Program
Division of Cancer Etiology
National Cancer Institute
Bethesda, Maryland, USA.

**References**

BRANDT-RAUF, P.W., PINCUS, M.R. & ADELSON, S. (1986). Carcinoma of the ampulla of Vater. *Dig. Dis.*, **4**, 43–48.

COHEN, J.R., KUCHTA, N., GELLER, N., SHIRES, G.T. & DINEEN, P. (1982). Pancreaticoduodenectomy: a 40-year experience. *Ann. Surg.*, **195**, 608–616.

JAGELMAN, D.G., DECOSSE, J.J. & BUSSEY, H.J.R. Upper gastrointestinal cancer in familial adenomatous polyposis. *Lancet*, **i**, 1149–1151.

LOVE, R.R. (1985). Small bowel cancers, B-cell lymphatic leukemia, and six primary cancers with metastases and prolonged survival in the cancer family syndrome of Lynch. *Cancer*, **55**, 499–502.

LOWENFELS, A.B. (1978). Does bile promote extra-colonic cancer? *Lancet*, **ii**, 239–241.

MECKLIN, J.-P., JARVINEN, H.J. & VIROLAINEN, M. (1992). The association between cholangiocarcinoma and hereditary non-polyposis colorectal carcinoma. *Cancer*, **69**, 1112–1114.

ROBERTSON, J.F.R., BOYLE, P. & IMRIE, C.W. (1988). Patients with ampullary carcinoma are prone to other malignant tumours. *Br. J. Cancer.*, **58**, 216–218.

SCHLIPPERT, W., LUCKE, D., ANURAS, S. & CHRISTENSEN, J. (1978). Carcinoma of the papilla of Vater. A review of fifty-seven cases. *Am. J. Surg.*, **135**, 763–770.

SCHLOSSBERG, D., WEBER, W., STOFFEL, U., HENGGELER, K., STOCKER, H., FOEPPL, M. & AKOVBIANTZ, A. (1988). Periampullary, colorectal, and gastric cancer in two siblings. *Int. J. Cancer*, **42**, 839–841.

SPIGELMAN, A.D., WILLIAMS, C.B., TALBOT, I.C., DOMIZIO, P. & PHILLIPS, R.K.S. (1989). Upper gastrointestinal cancer in patients with familial adenomatous polyposis. *Lancet*, **ii**, 783–785.